# Three-dimensional intracardiac ultrasound-guided catheter ablation for pregnancy-associated supraventricular tachycardia: a case report

**DOI:** 10.1186/s12884-026-09435-0

**Published:** 2026-06-08

**Authors:** Aoshuang Xing, Shuyu Yan, Ke Jing, Jin Jia

**Affiliations:** 1 Department of Obstetrics and Gynecology, West China Second University Hospital, Sichuan University, Chengdu, Sichuan 610041 China; 2 Key Laboratory of Obstetric & Gynecologic and Pediatric Diseases and Birth Defects of Ministry of Education, Chengdu, China

**Keywords:** Zero-fluoroscopy catheter ablation, Pregnancy-complicated supraventricular tachycardia, Case report, Multidisciplinary management

## Abstract

**Background:**

Physiological changes during pregnancy increase the risk of arrhythmias, with supraventricular tachycardia (SVT) being one of the most common types. Although often benign, SVT can cause symptoms such as palpitations and dyspnea, and in severe cases, may lead to hemodynamic instability, posing risks to both the mother and fetus. Managing SVT during pregnancy is challenging due to concerns about fetal safety with medications and the limitations of interventional procedures involving radiation. This report highlights the successful treatment of pregnancy-associated SVT using three-dimensional intracardiac ultrasound-guided radiofrequency ablation, ensuring excellent outcomes for both mother and baby.

**Case presentation:**

We report the case of a 35-year-old pregnant woman who successfully underwent zero-x-ray radiofrequency ablation (RFA) for left atrial tachycardia. At 12 weeks, sinus tachycardia was identified and treated with metoprolol, but symptoms and sustained atrial tachycardia persisted by 25 weeks. After multidisciplinary evaluation, zero-x-ray RFA was performed at 28 weeks for AVNRT, followed by a second RFA at 29 weeks to ablate tachycardia from the distal left atrial appendage. Post-procedure, symptoms resolved, and the patient delivered a healthy baby via elective cesarean at 37 weeks. Both mother and infant remained healthy at follow-up.

**Conclusion:**

Pregnancy complicated by SVT requires careful management due to the associated maternal and fetal risks. For patients with a history of SVT, catheter ablation prior to pregnancy is recommended to reduce recurrence and adverse outcomes. For pregnancy-onset SVT, early multidisciplinary consultation and individualized treatment are essential. Non-radiation radiofrequency ablation can be a safe and effective option in the second or third trimester to minimize fetal radiation exposure. Standardized, patient-centered management with multidisciplinary collaboration is crucial to ensuring maternal and fetal safety.

## Background

Physiological changes in pregnancy increase the risk of arrhythmias, with supraventricular tachycardia (SVT) being one of the most common types. SVT can occur at any stage of gestation, presenting with symptoms such as palpitations, dyspnea, and chest pain. In severe cases, it may cause hemodynamic instability, endangering both maternal and fetal health. Although SVT is generally considered benign during pregnancy, delayed diagnosis and treatment can lead to serious complications. Managing pregnancy-associated SVT is challenging due to concerns about fetal safety. Pharmacological therapy, while common, poses potential risks to the fetus, and interventional treatments like radiofrequency ablation are often limited because of radiation exposure. Therefore, individualized diagnostic and therapeutic strategies are essential to ensure maternal safety while minimizing fetal risks. This report presents a case of a pregnant woman with SVT successfully treated with catheter-based three-dimensional intracardiac ultrasound-guided radiofrequency ablation, achieving excellent outcomes for both mother and baby.

## Case presentation

**Fig. 1:**
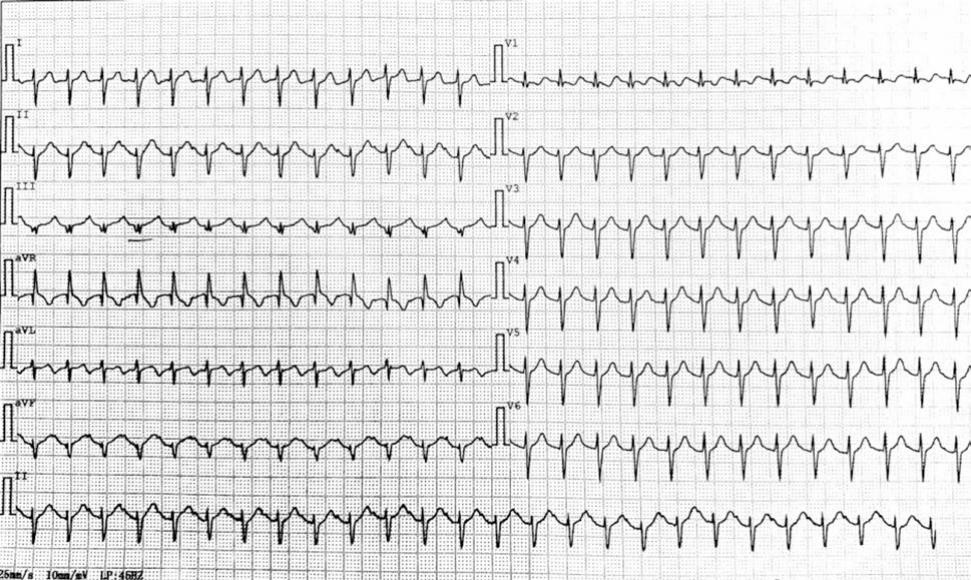
ECG at 12 weeks showed a narrow QRS tachycardia with P-wave morphology suggestive of left superior atrial tachycardia, with right axis deviation

**Fig. 2:**
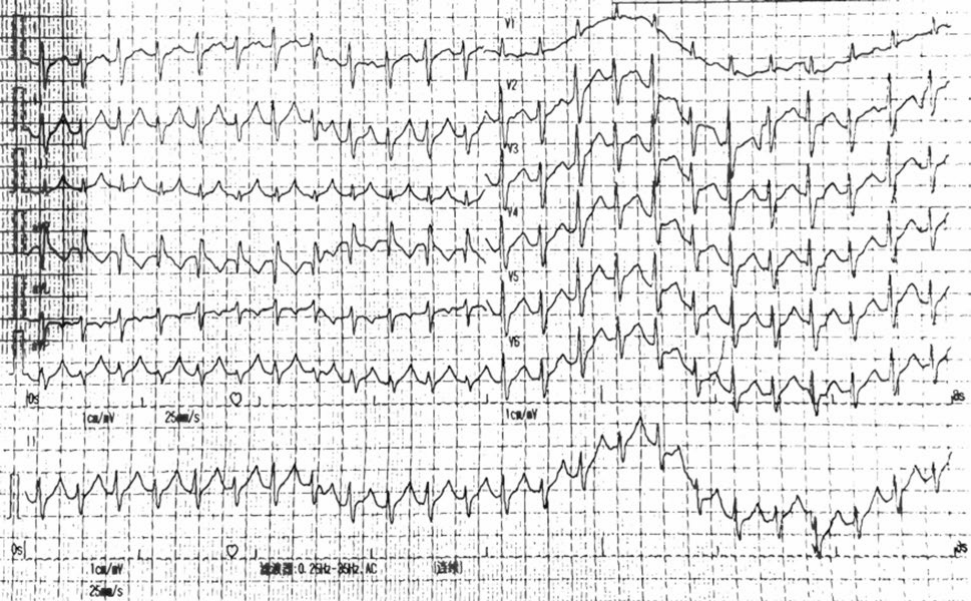
ECG at 25 weeks showed supraventricular tachycardia

At 25 weeks, the patient was admitted for palpitations and visual disturbances. She had a BP of 100/63 mmHg and irregular heartbeat (HR 100–180 bpm) without murmurs. Laboratory tests showed BNP 69.21pg/ml (< 100 pg/ml), Myoglobin 24.0 µg/ml (0–110 µg/ml), cTnI<3ng/L (0-39.59ng/L), CK 29U/L (39–192 U/L), CK-MB < 0.18 µg/L (0–5 µg/L), and elevated D-dimer at 1.11 µg/ml (< 0.55 µg/ml). Other parameters were normal. ECG confirmed supraventricular tachycardia (Fig. 2), while transthoracic echocardiography (TTE) revealed an LVEF of 66%, left atrial diameter of 36 mm (< 38 mm), interventricular septal thickness of 9 mm (8–15 mm), mild tricuspid regurgitation, and no structural abnormalities. Holter monitoring recorded 146,715 total heartbeats, with a mean HR of 108 bpm (75–198 bpm), 50,118 PACs, 161 VPCs, and 17,746 AEBs. Despite treatment with metoprolol and verapamil, episodes of tachycardia persisted, although symptoms partially improved. Adenosine administration (6 mg as a rapid intravenous bolus) resulted in transient termination of tachycardia; however, recurrent episodes were observed.

Given the complexity and potential risks associated with arrhythmia management during pregnancy, a multidisciplinary pregnancy heart team, including obstetricians, cardiologists, and electrophysiologists, was involved in the clinical decision-making process. The risks and benefits of medical therapy and catheter ablation were carefully discussed. Following comprehensive counseling, a shared decision-making process was conducted with the patient and her family, leading to the decision to proceed with catheter ablation. At 28 weeks of gestation, an electrophysiological study was performed under local lidocaine anesthesia with the patient in the supine position. The patient remained hemodynamically stable throughout the procedure, with no hypotension or clinical evidence of inferior vena cava compression. Despite repeated programmed stimulation, no sustained tachycardia was inducible. Based on preprocedural surface ECG characteristics and limited findings during the study, atrioventricular nodal reentrant tachycardia (AVNRT) was considered the most likely diagnosis, although definitive electrophysiological confirmation was not achieved. Given the risk of recurrent symptomatic tachycardia during pregnancy, an empirical modified slow pathway ablation was performed under intracardiac three-dimensional ultrasound guidance after informed consent had been obtained. Post-ablation electrophysiological testing demonstrated no inducible tachycardia or evidence of dual AV nodal physiology. However, tachycardia subsequently recurred, with ECG findings suggestive of atrial tachycardia. At 29 weeks, a second ultrasound-guided catheter ablation located and successfully ablated the tachycardia originating from the distal left atrial appendage. Postoperatively, the patient’s rhythm was stable, and symptoms were alleviated. At 33 weeks, follow-up Holter ECG showed sinus arrhythmia with an average HR of 98 bpm and no significant abnormalities. At 37 weeks, she delivered a healthy 3,240 g baby via elective cesarean section under combined spinal-epidural anesthesia, with Apgar scores of 10 at 1 and 5 min. The postoperative period was uneventful, and the patient was discharged 3 days later without symptoms. At one-month follow-up, both mother and infant were in excellent health. Written informed consent was provided for the publication of this case report.

## Discussion and conclusions

Pregnancy complicated by heart disease is typically classified into three categories in clinical practice: structural heart disease, functional heart disease, and pregnancy-specific heart disease. Functional heart disease primarily encompasses various arrhythmias that occur without underlying structural cardiovascular abnormalities, including both tachyarrhythmias and bradyarrhythmias. Tachyarrhythmias are one of the most common forms of heart disease during pregnancy and mainly include supraventricular arrhythmias (SVA) and ventricular arrhythmias. Pregnancy induces significant changes in maternal cardiac anatomy, hemodynamics, and hormonal levels, making it a high-risk period for the development of new arrhythmias or the recurrence of preexisting ones. Maternal blood volume increases by up to 50%, compensating for reduced systemic vascular resistance and supporting uteroplacental circulation [1]. The resulting stretch of the atria and ventricles, combined with physiological increases in heart rate, myocardial contractility, sympathetic activity, and altered catecholamine sensitivity, creates a highly arrhythmogenic state [2, 3]. Given the markedly increased susceptibility to arrhythmias during pregnancy, accurate rhythm identification is of critical importance. Standard ECG and Holter monitoring largely rely on automated algorithms for rhythm classification. Although such tools have value for screening, their discriminatory performance may be limited when distinguishing atrial tachycardia from sinus tachycardia or when differentiating other supraventricular arrhythmias. Therefore, manual review of ECG tracings is indispensable, particularly with careful verification of key electrophysiological features to ensure diagnostic accuracy and appropriate subsequent management.

Supraventricular arrhythmias (SVAs) are the most common form of arrhythmia during pregnancy, defined as tachycardia originating from the His bundle or higher structures (excluding atrial fibrillation) and characterized by atrial and/or ventricular rates exceeding 100 beats per minute [4]. The epidemiological data on pregnancy-related arrhythmias remain limited and somewhat controversial [5]. In China, studies estimate that 24–33 cases of supraventricular tachycardia (SVT) occur per 100,000 pregnancies [6, 7]. However, this figure may underestimate the actual incidence, as outpatient cases are often excluded from these statistics. The clinical presentation of SVT varies widely, including symptoms such as palpitations, shortness of breath, and hemodynamic instability, although some cases may be entirely asymptomatic [8, 9]. Research indicates that SVT not only raises the risk of severe maternal complications and cesarean delivery but is also associated with adverse pregnancy outcomes, such as low birth weight, premature birth, fetal distress, and congenital malformations [10].

Currently, the management of pregnancy complicated by SVT remains a significant challenge. In the acute onset of SVT during pregnancy, it is recommended to first attempt the Valsalva maneuver to terminate the tachycardia, thereby minimizing the potential impact of pharmacological interventions on both the mother and fetus. If unsuccessful, short-term use of antiarrhythmic medications may be considered. Adenosine, due to its short half-life, rapid metabolism, and minimal impact on the fetus, with a conversion success rate of up to 84%, is the drug of choice [8, 9]. In cases where adenosine is ineffective or contraindicated, intravenous administration of selective β-blockers (excluding atenolol) may be used to restore sinus rhythm or control ventricular rate [8, 11]. For patients with hemodynamic instability, immediate electrical cardioversion should be performed.

Long-term management of supraventricular tachycardia during pregnancy requires a careful balance between maternal and fetal risks and benefits. Efforts should be made to avoid antiarrhythmic drugs in the early stages of pregnancy, and the lowest effective doses should be used during the second and third trimesters. Commonly used antiarrhythmic medications can cross the placenta or be excreted in breast milk, and their efficacy and safety data are limited, lacking robust evidence from randomized controlled trials [12, 13]. In accordance with current clinical practice, β-blockers or verapamil are generally recommended as first-line agents for the management of supraventricular arrhythmias in the second and third trimesters in patients without pre-excitation. In the present case, the patient was initially treated with metoprolol, adenosine, and verapamil; however, despite these interventions, recurrent tachycardia episodes persisted, indicating suboptimal pharmacological control. Regarding class Ic antiarrhythmic agents, flecainide is not available in China, and propafenone is more commonly used in clinical practice [14]. However, the use of propafenone during pregnancy remains limited. Although available human data have not demonstrated teratogenic effects, propafenone can cross the placenta, and animal studies have suggested potential adverse fetal effects at high doses, which restricts its routine use in pregnancy [15, 16]. Catheter ablation, with its high success rate and favorable safety profile, may be considered as a treatment option in the second and third trimesters. Especially in experienced electrophysiology centers, three-dimensional electroanatomical mapping systems and intracardiac ultrasound techniques can facilitate low-radiation or even zero-fluoroscopy procedures [17–19].

For refractory SVT during pregnancy, particularly in cases where pharmacological treatment is ineffective or poorly tolerated, non-radiation radiofrequency ablation has demonstrated unique advantages. This technique can eliminate the pathological myocardial changes caused by arrhythmias while avoiding fetal radiation exposure, offering a high cure rate and stable efficacy [20, 21]. However, due to the lack of large-scale, multi-center clinical studies, the safety of non-radiation radiofrequency ablation during pregnancy has not been fully assessed. Although adverse event rates in existing literature are low, research on maternal and neonatal outcomes is still limited, and the potential long-term impact of this technique on prognosis cannot be ruled out. Therefore, large-scale cohort studies are urgently needed to explore the long-term safety and prognostic implications of this treatment.

The patient in this case presented with paroxysmal SVT on electrocardiogram. Despite treatment with adenosine and verapamil, the therapeutic effect was suboptimal, and the patient continued to experience intermittent palpitations and discomfort. After discussing with the patient, radiofrequency ablation was indicated, and the patient underwent electrophysiological testing under local anesthesia, followed by SoundStar intracardiac three-dimensional ultrasound-guided catheter radiofrequency ablation. During the first study, the inability to induce tachycardia meant that diagnosis and management had to rely primarily on clinical judgment. Clinical decision-making largely depended on the operator’s experience. Previous reports suggest that in a subset of patients with a high clinical suspicion of AVNRT, particularly when symptom burden is significant and the risk of recurrence is high, empirical slow pathway ablation may be considered [22–24]. This is also consistent with the management strategy used in this case. Following the second ablation procedure, the patient’s cardiac symptoms were significantly relieved, with a resting heart rate maintained at 80–90 beats per minute and stable hemodynamics. The patient continued regular antenatal check-ups and carried the pregnancy to 37 weeks, delivering a full-term infant via elective cesarean section, with good postoperative recovery.

In summary, according to current guidelines, for patients with a history of SVT who are planning pregnancy, it is recommended to undergo catheter ablation prior to pregnancy to cure the tachycardia, thereby reducing the risk of SVT episodes during pregnancy and avoiding adverse effects on both the mother and fetus. For patients presenting with arrhythmias for the first time during pregnancy, early multidisciplinary consultation is advised, involving cardiology, obstetrics, anesthesia, and other relevant departments. This approach helps determine the type, severity, and potential triggers of the arrhythmia, and an individualized treatment plan should be developed based on the patient’s specific situation. During the management process, a thorough history and cardiac examination should be conducted, with detailed inquiries about the patient’s arrhythmic episodes, including their characteristics, triggers, duration, frequency, and associated symptoms. Additionally, the presence of other cardiovascular diseases or pregnancy-related complications should be assessed to provide a comprehensive evaluation and guide integrated treatment. For patients who are candidates for radiofrequency ablation, non-radiation radiofrequency ablation may be chosen in the second or third trimester to minimize the potential radiation risk to the fetus. In conclusion, the standardized management of pregnancy complicated by SVT should prioritize patient safety, incorporating multidisciplinary collaboration and individualized treatment plans to minimize maternal and fetal risks and ensure the safety of pregnancy.

## Data Availability

Further details are available from the corresponding author.

## Abbreviations

SVT: Supraventricular Tachycardia
BMI: Body Mass Index
ECG: Electrocardiogram
BNP: Brain Natriuretic Peptide
cTnI: Cardiac Troponin I
CK: Creatine Kinase
CK-MB: Creatine Kinase-MB
HR: Heart Rate
TTE: Transthoracic Echocardiography
LVEF: Left Ventricular Ejection Fraction
PACs: Premature Atrial Contractions
VPCs: Ventricular Premature Contractions
AEBs: Atrial Ectopic Beats
RFA: Radiofrequency Ablation
AVNRT: Atrioventricular Nodal Reentrant Tachycardia
SVA: Supraventricular Arrhythmias
